# 

**Published:** 1874-08

**Authors:** 


### INDEX.

### A

Abstract of the Proceedings of the Buffalo Medical Association, 25, 66, 106, 120, 161, 252, 295, 327
A Case of Myelitis ending in Recovery.... 429
A Case of Worms in the Urinary Bladder, 431
Albany County Medical Society. Semi-Monthly Meetings. Reported by F. C. Curtis, M. D. ...11, 58, 92, 170
Albany Medical College, Meeting of the Alumni, ... 195
Alumni Association of the Medical Department of the University of Buffalo, ...194, 237
Alumni Association, Meeting of the, ... 314
American Medical Association, ... 354
American Medical Association and Medical Education ... 393
American Surgery, Prof. Erichsen on.... 230
Aneurism, Femoral, Cure of, Treated first by Compression and subsequently by ligature of the External Iliac. By C. C. F. Gay, ... 344

### B

Bartow, Bernard, M. D. Report of Surgical Cases in the Buffalo General Hospital with Clinical Remarks by Prof. J. F. Miner, M. D. ... 186, 285
Bennett, Wm. A., M. D. Is Phthisis Hereditary? ... 381
Bissell, Daniel P. M. D. Obituary, ... 157
Blackham, Geo. E., M. D. Trichina Spiralis and Trichiniasis, ... 241
Blistering. By E. T. Dorland, M. D, ... 452
Blood. To what are the Changes in the Color due. By E. M. Moore, Jr., M. D.. 126
Books and Pamphlets Received, ... 40, 80, 120, 160, 200, 240, 280, 320, 360, 400, 440, 474
Brown, J. N., M. D. Obituary, ... 75
Brush, Edward N., M. D. Notes on Obstetrics and Gynæcology, ... 389, 458
Brush, Edward N., M. D. Syphilitic Affections or the Nervous System, ... 81, 130
Buffalo General Hospital ... 396
Buffalo Medical Association, Abstract of the Proceedings of ... 25, 66, 106, 120, 161, 252, 295, 327
Buffalo Medical College. Preliminary Term in, ... 34
Buffalo Medical College. Twenty-Ninth Annual Commencement, ... 277

### C

California, The Medical Profession in, ... 434
Camp Diarrhœa. By T. M. Johnson, M. D. 330
Causes of Disease in large Cities, ... 74
Cerebro-Spinal Anæmia and Convulsions. By W. W. Munson, M. D. ... 281
Chapman, E. N., M. D. The true Uterine Mucous Membrane, its Structure, etc.... 361
Children, The Care of the, ... 473
Cincho-Quinine, ... 149
Clinical Remarks upon Surgical Cases occurring in the Buffalo General Hospital. By Prof. J. F. Miner, M. D. ... 186, 285
Clinical Remarks upon Surgical Cases occurring in the Hospital of the Sisters of Charity. By Prof. J. F. Miner, M. D.... 113, 135, 181, 226, 308, 321
Colles Fracture, Ununited, Resection of a portion of the Ulna and Radius for, ... 353
Commencement. Twenty-Ninth Annual of the Buffalo Medical College, ... 277
**CORRESPONDENCE.**—
A New Medical Law, ... 33
Hemorrhoidal Tumors, ... 139
Inversion of the Uterus, ... 410
Report of Cases in the Public Prints, ... 118
Coventry, Prof. Charles B. Obituary, ... 317
Curtis, F. C., M. D. Report of the Transactions of the Albany County Medical Society, ... 11, 58, 92, 170

### D

Death following Vaginal Injection, ... 193
Discussion on the Use of Digitalis, Chloral and Bromide of Potassium, ... 151
Disease in large Cities, Cause of ... 74
Dorland, E. T., M. D. Blistering, ... 452

### E

Education, Medical, and the American Medical Association, ... 393
Education, Preliminary, and the Erie County Medical Society, ... 397
Education, Preliminary, ... 469
Erichsen, Prof., On American Surgery, ... 230
Erie County Medical Society, ... 196
Erie County Medical Society, Annual Meeting of ... 233

### G

Gay, C. C. F., M. D. Femoral Aneurism, Cure of, Treated first by Compression, subsequently by ligature of the External Iliac, ... 344
Gay, C. C. F., M. D. Injuries of the Skull, their relation to Medical Evidence, with report of Cases, etc., ... 1
Gay, C. C. F., M. D. Preventive Medicine, 441
Grave Robbing, Recent in the City, ... 117

### H

Homeophathic Physicians and Regular Medical Societies, ... 235
Hospital Sunday, ... 33

### I

Injuries of the Skull, their relation to Medical Evidence, with report of Cases and Remarks upon the Employment of the Trephine. By C. C. F. Gay, M. D. ... 1
Inversio-Uteri. Report of a Case, ... 349

Iodide of Potassium the Therapeutic value of........................................ 312
Ipecacuanha. Some Observations on the Local action of........................ 147
Ipecacuanha Spray. The use of, in Winter Cough and Bronchitic Asthma,............ 141
Is Phthisis Hereditary? By Wm. A. Bennett, M. D.,.............................. 381
Items.....36, 76, 239, 278, 315, 356, 400, 435, 473

J

Johnson, T. M., M. D. Camp Diarrhœa and Dysentery,............................ 330

M

Medical Education.............................. 393
Medical Literature.............................. 355
Medical Society of the State of New York, Sixty-ninth Session.................. 262
Mercer, Alfred, M, D. The Relations of General Medicine to Specific Modes of Medication.................................... 41
Miner, Prof, J. F., M. D. Clinical Remarks upon Surgical Cases at the Buffalo General Hospital..........................186, 285
Miner, Prof. J. F., M. D. Clinical Remarks upon Surgical Cases at the Buffalo Hospital of the Sisters of Charity,.......... 113, 135, 181, 226, 308, 321
Miner. Prof. J. F., M. D. Ovariotomy by Enucleation, what it is and how to do it 401
Miner, W. W., M. D. Report of Clinical Remarks by Prof. J. F. Miner.......... 113, 135, 181, 186, 226, 285, 308, 321
Moore. E. M., Jr., M. D. To what are the changes in the color of the Blood due?...126
Munson, W. W., M. D. Cerebro-Spinal Anæmia and Convulsions.................. 281
Myelitis, a case ending in recovery......... 429

N

Nervous Accidents of Syphilis. By John B. Stonehouse, M. D...................... 201
Nervous System, Syphilitic Affections of the By Edward N. Brush, M. D.....81, 130
New York State Medical Society, Sixty-ninth Session............................ 262
Note on Salicylic Acid...................... 337
Notes on Obstretics and Gynæcology. By E. N. Brush, M. D................389, 458
Notice to our Subscribers.................... 119

O

**OBITUARY.—**
Daniel P. Bissel, M. D.......... 157
Jeremiah N. Brown, M. D........ 75
Prof. Charles B. Coventry, M. D. 317
Prof. Jeffries Wyman, M. D..... 76
Rev. Asher Wright.............. 356
Ohio State Medical Society.................. 415
Ovariotomy by Enucleation. What it is and how to do it. By Prof. J. F. Miner, M. D............................................ 401

P

Phelps, Wm. C., M. D. Tracheotomy in Croup...................................... 405
Puerperal Fever, Precautions with regard to,.......................................... 468
Preston, B. J., M. D. Syphilitic Hydrosarcocele...................................... 408
Preventive Medicine. By C. C. Gay, M. D. 441
Public Health................................ 190

R

Relation of the Profession to the Secular Press and the Rostrum.................... 420
Report of a Case of Inversio Uteri........ 349
Resection of a portion of the Ulna and Radius for Ununited Colles Fracture...... 353

**REVIEWS.—**
A Guide to the Practical Examination of the Urine. By James Tyson, M. D...................... 198
A Handbook of Medical and Surgical Reference. By John H. Wyeth, M. D...................... 40
A Hand-book of Obstetric Surgery. By Charles Clay, M. D.......... 119
A Practical Treatise on Diseases of Women. By T. Gaillard Thomas, M. D............................. 196
Archives of Dematology. Edited by L. D. Buckley, M. D.......... 159
Archives of Electrology and Neurology. Edited by Geo. M. Beard, M. D............................. 39
A Treatise on Diseases of the Genito-Urinary Organs and Syphilis. By W. H. Van Buren, M. D., and E. L. Keyes, M. D...... 79
A Treatise on Food and Dietetics. By F. W. Pavy, M. D., F. R. S.. 38
A Treatise on Theapeutics, Comprising Materia Medica and Toxicology. By H. C. Wood, Jr., M. D.......................... 37
Braithwait's Retrospect.............. 39
Clinical Lectures. By N. S. Davis, M. D............................ 198
Clinical Lectures on Diseases of the Nervous System. By Wm. H. Hammond, M. D................ 200
Compendium of Children's Diseases. By Johann Steiner, M. D. 358
Conspectus of Medical Sciences. By E. H. Hartshorne, A. M., M. D.............................. 159
Croup in its Relations to Tracheotomy. By J. Solis Cohen, M. D. 317
Cyclopædia of the Practice of Medicine. Edited by Dr. H. Von Ziemssen. Vol. I.............. 279
Cyclopædia of the Practice of Medicine. Edited by Dr. H. Von Ziemssen. Vol. II............. 358
Dental Pathology and Surgery. By S. James A. Salter, M. D., F. R. S. 360
Diseases of the Stomach. By Wilson Fox, M. D................ 359
Erysipelas and Child-bed Fever. By Thomas C. Minor, M. D..... 318
Essentials of the Principles and Practice of Medicine. By Henry Hartshorne, M. D............. 239
Lectures on Diseases of Infancy and Childhood. By Charles West, M. D............................ 38
Lectures on Diseases of the Respiratory Organs, Heart and Kidneys. By A. L. Loomis, M. D... 439
Lindsay and Blakiston's Visiting List............................. 199
Materia Medica for the use of Students. By John B. Biddle, M. D. 158
Observations on the Pathology and Treatment of Cholera. By John Murray, M. D................. 39
Orthopædia. By James Knight, M. D............................ 437
Outlines of the Science and Practice of Medicine. By Wm. Aitken, M. D., F. R. S............ 319
Pulmonary Tuberculosis. By Addison P. Dutcher M. D......... 32

Surgical Emergencies. By Wm. P. Swain, M. D .... 319
Syphilitic Lesions of the Osseous System in Infants and Young Children. By R. W. Taylor, M. D. .... 438
The Building of a Brain. By E. H. Clark, M. D .... 158
The Breath, and Diseases which give it a Fetid Odor. By J. W. Howe, M. D .... 199
The Histology and Histo-Chemistry of Man. By Heinrich Frey .... 437
Therapeutics and Materia Medica. By Alfred Stille. M. D .... 197
The Physician's Dose and Symptom Book. By Joseph Wyethes, M. D .... 40
The Physiology of Man. Vol. V. By Austin Flint, Jr., M. D .... 77
The Virginia Medical Monthly. Edited by Landon B. Edwards, M. D .... 199
Rochester Medical Society, Semi-monthly Meeting of .... 73, 174
Roe, J. O., M. D. Report of Semi-monthly Meeeting of the Rochester Medical Society .... 73, 174

**S**

Salicylic Acid, Note on .... 337
Some Observations on the Local Action of Ipecacuanha .... 147
Stonehouse, John B., M. D. The Nervous Accidents of Syphilis .... 201
Subscribers, Notice to .... 119
Subscribers, To our .... 195
Sugar-coated Pills .... 398
Syphilis, The Nervous Accidents of. By John B. Stonehouse, M. D. .... 201
Syphilitic Hydro-sarcocele. By B. J. Preston, M. D .... 408
Syphilitic Meningitis and Cerebral Disease 155

**T**

Taste, The Sense of .... 431
The Condition of the Uterus five weeks after Delivery .... 158
The New York Observer .... 195
Therapeutive Value of Iodide of Potassium 312
The Relations of General Medicine to Specific Modes of Medication. By Alfred Mercer, M. D .... 41
The State Legislature and Vivisection .... 236
The True Uterine Mucous Membrane, its Structure, Functions, and Morbid States. By E. N. Chapman, M. D .... 361
The Use of Ipecacuanha Spray in Winter Cough, etc .... 141
To our Subscribers .... 195
To what are the Changes in the Color of the Blood due? By E. M. Moore, Jr., M. D .... 126
Tracheotomy in Croup. By Wm. C. Phelps, M. D .... 405
Transfusion .... 433
Trichina Spiralis and Trichiniasis. By Geo. E. Blackham, M. D .... 241

**U**

Uterus, Filroids of the .... 433
Uterine Mucous Membrane, its Structure, Functions, and Morbid States. By E. N. Chapman, M. D .... 361
Uterus, The Condition of the, five weeks after Delivery .... 153

**V**

Vaginal Injections, Death following .... 193
Vivisection. .... 193
Vivisection and the State Legislature .... 236
Volume Fourteen .... 35

**W**

Warner, Wm. R. Co.'s Pills .... 35
Worms in the Urinary Bladder, A Case of. 431
Wright, Rev. Asher. Obituary. .... 356
Wyman, Prof. Jeffries, M. D. Obituary .... 76

### Books and Pamphlets Received.

The Physiology of Man; designed to represent the Existing State of Physiological Science as applied to the Functions of the Human Body. By Austin Flint, Jr., M. D., etc. Vol. V. Special Senses; Generation. New York: D. Appleton & Co., 1874. Herger & Ulbrich.

Essays on Conservative Medicine and Kindred Topics. By Austin Flint, M. D., Prof., etc. Philadelphia: H. C. Lea, 1874. Buffalo: T. Butler & Son.

Transactions of the Medical and Chirurgical Faculty of the State of Maryland at its seventy-fifth Session. Baltimore, April, 1874.

The Toner Lectures. Lecture No. III, On Strain and Overaction of the Heart. By J. M. Da Costa, M. D., etc. Delivered May 14, 1874. Washington: Smithsonian Institution, 1874.

Address of Joseph M. Toner, M. D., President of the American Medical Association at Detroit, June, 1874.

Nomenclature of Diseases prepared for the use of the Medical Officers of the U. S. Marine Hospital Service. By John M. Woodworth, M. D., Supervising Surgeon. Washington: 1874.

The Relation of Medical Societies to Progress in Science. Inaugural Address of the President of the Kings Co., N. Y., Medical Society, Alex. J. C. Skene, M. D. June 16, 1874.

List of the Officers and Members of the American Social Science Association for 1874.

Peters' Musical Monthly, Vol. XIV, Nos. 83, 84 and 85. New York: J. L. Peters, 559 Broadway.

